# Efficacy of Hypnosis on Dental Anxiety and Phobia: A Systematic Review and Meta-Analysis

**DOI:** 10.3390/brainsci12050521

**Published:** 2022-04-20

**Authors:** Thomas Gerhard Wolf, Sina Schläppi, Carla Irene Benz, Guglielmo Campus

**Affiliations:** 1 Department of Restorative, Preventive and Pediatric Dentistry, School of Dental Medicine, University of Bern, CH-3010 Bern, Switzerland; sina.schlaeppi@students.unibe.ch (S.S.); guglielmo.campus@zmk.unibe.ch (G.C.); 2 Department of Periodontology and Operative Dentistry, University Medical Center of the Johannes Gutenberg-University Mainz, D-55131 Mainz, Germany; 3 Department of Prosthodontics and Dental Technology, Faculty of Health, Witten-Herdecke University, D-58455 Witten, Germany; carla.benz@uni-wh.de; 4 Department of Surgery, Microsurgery and Medicine Sciences, School of Dentistry, University of Sassari, I-07100 Sassari, Italy

**Keywords:** dental anxiety, fear, dental phobia, effect, hypnosis, hypnotherapy

## Abstract

Hypnosis is a commonly used therapy option in dentistry and medicine for fear and pain reduction. Nevertheless, it is viewed very critically, as there is still insufficient evidence for a treatment effect. Specific phobia of dental treatment and dental anxiety are prevalent conditions that can cause an oral health impairment. This paper critically reviews 19 clinical trials aimed at reducing dental anxiety and fear avoidance in adults, published in peer-reviewed journals between 1979 and 2021. The search identified 257 papers; 223 were selected after removing duplicates. A total of 188 articles were excluded after title and abstract evaluation; 35 full text articles were assessed for eligibility. Another 10 papers were discharged after full text evaluation, as these were case reports and questionnaires. Six papers were discharged due to the lack of a comparable scale to measure dental anxiety. The following treatment techniques were reviewed: various forms of cognitive-behavioral therapy (CBT), relaxation training, benzodiazepine premedication, self-hypnosis by audio therapy, hypnotherapy, hypnosis, and nitrous oxide sedation. CBT delivered in a variety of formats, including one-session treatment, showed the most evidence for the efficacy of reducing anxiety. A wide heterogeneity of methods allowed only the inclusion of five studies to the performed meta-analysis, showing contrasting results for the application of hypnosis. The main reason for this issue is the great variety in methods used, making a distinct assessment of hypnotic interventions difficult. However, the results of the systematic review are promising in that hypnosis can also be regarded as powerful and successful method for anxiety reduction, while there are also studies with a small or even slightly negative effect. Therefore, further research is needed. Within the limitations of the current study, a more consistent use of methods to examine anxiety for hypnosis research is recommended.

## 1. Introduction

Neuroscientific evidence interprets hypnotic trance as a modified state of consciousness that accentuates concentration, attention, and the setting free of thoughts [1,2]. The American Psychological Association (APA) Division 30 defines hypnosis as “a state of consciousness involving focused attention and reduced peripheral awareness characterized by an enhanced capacity for response to suggestion” [3].

The phenomenology of hypnosis can be considered as the result of intentional introspective activity; it enables the patient to improve control over his mind and body, the main effects being hypnotic analgesia and reduction of anxiety [4].

Dental anxiety or dental phobia must be differentiated. Dental phobias show severity of their physiological and psychological symptoms. Triggers of fear are the perception of the visual, olfactory, and sensory stimuli during dental treatment (sound of drill, dentist’s chair) [5]. Good cooperation/rapport is necessary. Dental procedures are often carried out under sedation or general anesthesia in order to achieve adequate treatment conditions for both doctor and patient. Regardless of all dental progress, one of the major challenges of treating patients is the management of pre- and intraoperative anxiety of the patient [3]. Therefore, another option for treating patients with dental phobia is needed [3]. Visualization, suggestion, and hypnosis strategies train the patients to put themselves on a level of focused consciousness so that suggestions can be easily accepted. Regardless of the technique used, its effectiveness depends heavily on how it is applied, including the empathic abilities demonstrated by the dentist [6].

The good acceptance of hypnosis and the effectiveness in reducing anxiety in patients with dental phobia is reported by several authors [5,7,8,9,10,11]. Positive effects of hypnosis in patients with dental phobias include reduction of fear and anxiety [5], prevention of avoidance behavior and the resulting lack of dental treatment, reduction of felt pain, less bleeding during tooth extractions, and better and faster wound healing [5,7,8,9,10,11].

The present paper was aimed to provide a comprehensive overview of hypnosis interventions for patients with severe levels of anxiety and dental phobia that are implementable in general dental practice before or during dental procedures. As an ancillary aim, the quantification of the efficacy of non-pharmacological intervention to reduce dental anxiety in patients undergoing dental procedures in comparison to standard care alone or to attention-control groups was also evaluated. To fulfill these aims, a systematic review with meta-analysis of the data was performed.

## 2. Materials and Methods

### 2.1. Review Design

**Figure 1:**
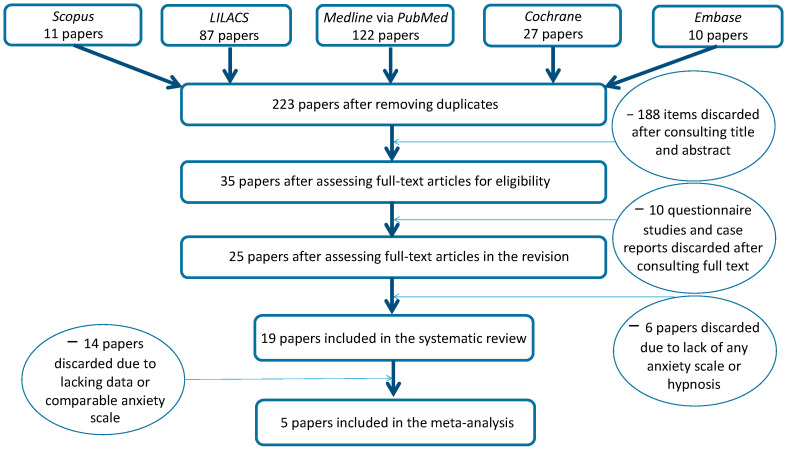
Flowchart of the study.

This systematic review follows the Preferred Reporting Items for Systematic Reviews and Meta-Analyses (PRISMA) guidelines [12] (Supplementary Table S3. PRISMA checklist). The review protocol was registered with the international prospective register of systematic reviews (PROSPERO) system on 14 July 2020 with ID-CRD42020172052. The flowchart of the study is depicted in Figure 1. 

### 2.2. Eligibility Criteria

Randomized controlled trials (RCTs), cross-sectional studies, comparative studies, validation studies and evaluation studies, reporting hypnosis effects for dental anxiety and phobia in patients of any age were searched and evaluated. Only papers in English published from the 1st of January 1979 to the 31 of December 2021 were collected.

### 2.3. Information Sources

Electronic databases (*Scopus, LILACS, Medline* via *PubMed, Cochrane,* and *Embase*) were screened for articles without any further restriction regarding language. Additionally, grey literature was retrieved (https://www.greynet.org, accessed on 10 April 2022).

### 2.4. Search Strategy

As a search scheme, we first tried to follow the PICO/PECO model [13] with the following indications:

***P*** *roblem:*

What is the effect of hypnosis for dental anxiety and dental phobia?

***E*** *xposure/**I**ntervention:*

Which measuring devices and procedures are used for this? Is there a standard device/measure for detection?

***C*** *ontrol:*

Is there a control group without hypnosis?

***O*** *utcome:*

Results.

However, there are not corresponding search terms for every indication. For example, there is no standard method to prove hypnotic effects, and the results are not clearly definable for this overview because different types of hypnosis show different results, especially concerning the brain areas. Therefore, in the areas where this was possible, research was generally done with matching keywords. The search strategy included a combination of MeSH terms and key words: dental anxiety OR dental fear OR dental phobia, OR hypnosis OR hypnotic OR dental hypnosis OR hypnotherapy OR sedation.

### 2.5. Study Selection

Repeated or duplicate papers were excluded after completing the search string comparing the results using the five databases. All authors independently examined all abstracts of the papers. All papers meeting the inclusion criteria were obtained in the full-text format. The authors independently assessed the papers to establish whether each paper should or should not be included in the systematic review.

### 2.6. Data Collection, Summary Measures, and Synthesis of Results

Data collection was carried out using an ad hoc designed data extraction form without masking journal title or authors. Data were extracted and synthesized by three authors (G.C., T.G.W., and S.S.) independently. For each paper, the following data were searched and recorded when available: (a) publication year and study duration; (b) details/characteristics of the participants at baseline; (c) hypnotherapy data, including actual dental fear survey/scale such as Dental Anxiety Scale, Visual Analog Scale, modified Dental Anxiety Scale, Spielberger State-Trait Anxiety Inventory, or Harvard group scale of Hypnotic Susceptibility; and (d) the presence of hypnotherapy or hypnosis. For the primary outcome, data from different studies were compared on the use of different scales to evaluate the effect of hypnosis to treat patients with dental anxiety and dental phobia. To facilitate the data synthesis, the results were summarised in tables where each selected paper was included, and the main aspects presented. Results were analyzed considering the following subgroups: adults with dental phobia vs. adults without dental phobia and dental treatment with hypnosis or hypnotherapy or without any hypnosis.

The meta-analysis of the data was carried out using the ProMeta 3 Software (IdoStatistics, https://idostatistics.com/prometa3/, accessed on 19 April 2022, Cesena, Italy). Odds ratio (OR) and mean difference (MD) were chosen for calculating effect size. The I^2^ statistic was calculated to describe the percentage of variation across studies due to heterogeneity rather than chance [14]. Due to the heterogeneous methods of the studies included in the systematic review and due to missing or incomplete data documentation, especially using scales such as the Dental Anxiety Scale in several studies, the meta-analysis could only be performed with studies that all used the State-Trait Anxiety Inventory (STAI) [7,15,16,17,18,19]. Therefore, further subgroups could not be created. The heterogeneity was categorized as follows: <30% not significant; 30–50% moderate; 51–75% substantial; and 76–100% considerable. Whether homogeneity was obtained or not, the random effects model (REM) with 95% confidence intervals was chosen as the meta-analysis model. The significance levels of the effect sizes were determined based on the two-tailed test. In all tests, the level of significance was set at *p* < 0.05.

### 2.7. Assessment of Bias across Studies

The risk of bias assessment was conducted by three authors (G.C., T.G.W., and S.S.). The methodological quality of the included RCTs was scored according to the customized quality assessment tool developed by the National Heart, Lung, and Blood Institute and Research Triangle Institute International for systematic reviews and meta-analyses, observational cohort and cross-sectional studies, pre-post studies with no control group and case series studies, as well as the accompanying study quality assessment tools guidance for assessing the quality of controlled intervention studies (www.nhlbi.nih.gov/health-topics/study-quality-assessment-tools, accessed on 19 April 2022). Criteria for appropriate randomization, participation rate, group/population similarity, adherence to intervention protocols, and sources of bias (publication bias, eligible subjects, exposure measurements, blinding, validity, selection bias, information bias, etc.) comprised quality control. A “yes/no/cannot be determined” selection was designated for each part. If the study was at the lowest risk for bias, each study/paper was rated good; if the study was predisposed to some bias, it was rated as moderate; and if there was a possibility that the study was biased, it was rated as poor.

## 3. Results

The search strategy identified 257 papers in the five databases; 223 were selected after removing duplicates. A total of 188 articles were excluded after title and abstract evaluation; 35 were assessed by full text, and 25 were full text assessed for eligibility after removing 10 papers of questionnaire studies and case reports. Another six papers were discharged after full text evaluation, as these were papers without hypnotherapy or parameters for measure anxiety reduction; the quality assessment scores of the papers included are presented in Table 1. (Supplementary Table S1: Details of included studies; Supplementary Table S2. List of excluded papers). Regarding reducing dental anxiety by using hypnosis, 15 papers were ranked of as being of good quality, 4 were classified of fair quality, and none was classified of poor quality. Details of included studies with authors, type of study, location, number of subjects/patients, sex, time of implementation, type of dental treatment, number of groups studied, and main results are depicted in Supplementary Table S1. Included studies with general characteristics are shown in Table 1.

### 3.1. Measurement of Anxiety Reduction

A total of 15 different methods were used to record the dental anxiety or dental fear.

### 3.2. DAS (Dental Anxiety Scale)

In this review, the DAS is the most utilized anxiety scale. Twelve studies using the Dental Anxiety Scale have been published [9,10,15,16,17,23,24,25,29,30,31]. This result reflects the theory that the DAS is generally the most-used scale in dentistry to measure anxiety.

### 3.3. Visual Analogue Scale (VAS)

Visual Analogue Scale (VAS) is a psychometric measuring instrument used to document and compare the characteristics of disease-related symptom severity in individual patients. A Visual Analogue Scale is usually a 100 mm long horizontal line with verbal descriptors (word anchors) at each end to express the extremes of feeling and the subjective strength of expression of certain symptoms. When the VAS is read, the position of the patient’s cross is generally given a value between 0 and 100 [32]. In this systematic review, nine of all included studies evaluated dental anxiety as measured by the Visual Analogue Scale [7,9,16,18,24,25,28,31,32].

### 3.4. STAI—State-Trait Anxiety Inventory

In seven of all included studies, the STAI (State-Trait Anxiety Inventory) verified the level of dental anxiety [7,15,16,17,18,19,23]. The included studies involved dental procedures such as endodontic treatment, tooth filling, tooth removal, removal of third mandibular molars, oral surgery, or dental treatment showed in a video. Five studies on tooth extraction under hypnosis have also been published [7,9,18,25,28].

### 3.5. Technique for Hypnosis

Different types of hypnosis and hypnotherapy were emphasized, such as hypnosis with an audiotape, hypnosis as progressive muscle relaxation, cognitive behavioral therapy (CBT), or standardized optical hypnosis method Chiasson’s technique. No meaningful differences have been observed when characteristics of hypnosis, group therapy as hypnosis by an audiotape, and individual systematic desensitization were compared [15]. The most common hypnotic technique used in the studies was the standardized hypnosis by using an audiotape. As seen in our review, it becomes the simplest and most used method for anxiety reduction using hypnosis. Self-hypnosis by an audiotape can be performed either live or by means of sound recording. For this purpose, the patient receives a sound carrier either individually or generally designed. In ten articles [9,10,16,17,18,22,23,25,26,28], the experimental group used an audio recording as self-hypnosis to reduce dental anxiety prior to surgery or dental treatment. Seven papers showed a significant effect of dental anxiety reduction by using an audiotape. One article showed only a marginal effect of anxiety reduction [23]. This preoperative hypnotic technique using a tape recording can achieve anxiety reduction, but further studies with more subjects are needed for statistical validity [28]. One article [17] suggests that CBT is the treatment of choice for dental phobia patients when comparing the effectiveness and acceptability of CBT, standard hypnosis, individualized hypnosis, and general anesthesia.

### 3.6. Anxiety Reduction by Using Hypnosis

The effectiveness of hypnosis is discussed in various studies. This literature review proves that hypnosis shows a positive effect on reduction of dental fear. Hypnosis used for dental anxiety has often not only marginal effect, as in these two studies [23,27], but it also shows a meaningful and significant effect for anxiety reduction during dental treatment [7,9,15,16,18,30]. One paper [22] underlines the decrease of the average diastolic blood pressure and heart rate during surgery during hypnotherapy by an audio pillow with relaxation music. In the control group without hypnosis, these parameters increased. Only one paper [7] examined showed no significant reduction of dental anxiety by using hypnosis. The results were examined using the STAI. That hypnosis sustains an effect on brain activity was shown in the study by Halsband and Wolf [5]. In a group of patients with dental phobias, the main effects of anxiety were found in the left amygdala and bilaterally in the anterior cingulate cortex (ACC), insula, and hippocampus (R < L). During hypnosis, the phobic subjects showed significantly reduced activation in all these areas. Reduced neural activity patterns were also observed in the control group. No amygdala activation was detected in healthy subjects under either of the two experimental conditions. Compared to the phobic subjects, the control group showed a lower bilateral activation in the insula and in the ACC in the waking state. The results suggest that anxiety-inducing stimuli, such as dental surgery, endodontic treatment, or inadequate anaesthesia, under hypnosis can be effectively reduced. This study provides scientific evidence that hypnosis is an effective and successful method to inhibit the fear reaction in the brain.

Details of the included studies with authors, type of study, location, number of subjects/patients, sex, time of implementation, type of dental treatment, number of groups studied, and main results are depicted in Table S1.

**Figure 2:**
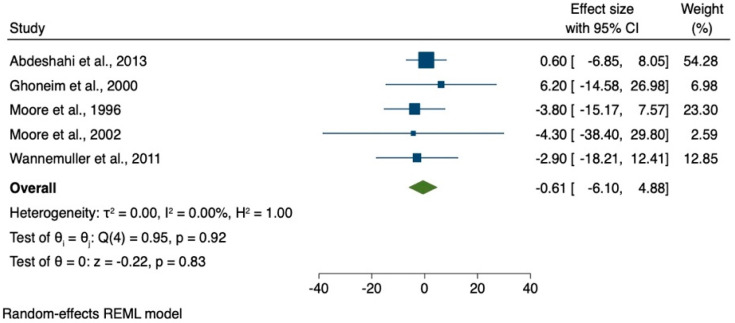
Meta-analysis of studies using State-Trait Anxiety Inventory (STAI).

The results of the meta-analysis of only five studies using the State-Trait Anxiety Inventory (STAI) are shown in Figure 2.

Due to the low numbers of studies included in the data synthesis for the meta-analysis, a high grade of heterogeneity was observed. The effect size ranges from −4.30 [15] to 6.20 [18].

## 4. Discussion

The present study aimed to systematically review studies about effects of reducing dental anxiety by using hypnosis during or prior to the dental treatment. In this review, a comprehensive systematic search was performed on studies that reported and evaluated the effect of hypnosis in dental anxiety and phobia in adults. Different types of questionnaires and test parameters to prove an anxiety reduction were exploited. In addition, there is no standard hypnosis therapy, but there are various ways to experience the hypnotic trance state and thus the reduction of anxiety. The results were mostly heterogeneous, probably due to the study design, the sampling methods, the application of the questionnaire, and the settings as well as cultural attitudes and socio-economical variations. Comparing hypnotic effects is difficult without any interindividual standard parameter measuring anxiety and fear. Parameters such as suggestibility or previous experience with hypnosis have an additional influence on the effect and thus the reduction of anxiety of the patient. Randomized clinical studies are difficult to conduct because the patient has individual needs for dental treatment, and there is no possibility to perform a double-blind study. Nevertheless, it is difficult to prove a statistically significant effect since the measurable parameters are derived from subjective perception. As already mentioned, a total of 15 different methods were used to measure anxiety. The Dental Anxiety Scale and the Visual Analogue Scale (VAS) are used to obtain a subjective assessment of the patient on paper. In addition, data from the same patient on both scales can differ slightly and can therefore not be compared one-to-one.

A limitation of our review is the quantitative measurement of dental anxiety, which has not been carried out with a questionnaire specifically designed for this purpose. Two included studies have been conducted in children [20,31]; here, it should be considered that children tolerate only a very short treatment time in the dental chair. The data in questionnaires should be interpreted very cautiously since children often do not yet have the cognitive abilities to make assessments, and the questionnaires are often completed together with the parents, who can have a strong influence on the data. Another limitation in the comparison of anxiety reduction using hypnosis is that not every study included anxiety measurement before and after dental treatment, as in the paper of Wannemueller et al. [17]. The patients were treated by standardized hypnosis; no significant advantage was found regarding dental treatment and anxiety reduction although the measured values did show a similar improvement as in the group with individualized hypnosis [17]. The results and statements are therefore questionable; however, in both groups, a great similarity in anxiety scores was observed [17]. The comparison of each subject with each other showed no significant differences; hypnosis did not increase the anxiety of the patients, but there was no significant anxiety reduction either [17]. 

In the study of Abdeshahi et al. [7] should be noted that the test and control procedures in this study were performed on the same patient, as one wisdom tooth was extracted under hypnosis, and the contralateral wisdom tooth was extracted in a second session without hypnosis. Thus, after the first hypnotic wisdom tooth extraction, the patient may have had a positive experience that altered the conditions for the second session or a possibly increased anxiety about the second extraction due to a negative experience [7]. As early as 1997, it was shown that hypnosis therapy for reducing dental anxiety may be effective [28]. This conclusion of the authors for reducing anxiety by hypnosis can be understood: the rather low significance may be due to the randomization, the low drop-out rate, the partial blinding, and the number of patients. 

A further limiting aspect of our review is the heterogeneity of hypnosis therapies. Probably the easiest therapy to implement and most frequently used method is the audio procedure, which does not require a trained hypnotist and is easily applicable for general dentists. This can be done with commercial audiotapes that can be used to achieve an anxiolytic effect. The audiotape enables standardization and reproducibility, which is important for scientific purposes [19]. A disadvantage is the lack of active psychological support and individual adaptation of hypnosis to the dental treatment situation. Depending on the patient’s structure, the pre-formulated and unchangeable contents and suggestions of the audiotape could be perceived as too low or too directive [19], resulting in resistance of the patient against suggestions. Although individual live hypnosis needs more effort compared to audiotape, the therapeutic effectiveness of live hypnosis is higher than hypnosis by audiotape [33].

It is important to investigate the effectiveness of hypnotic strategies by further randomized clinical studies with regular group homogeneity and detailed comprehensible data sets as well as adequately collected measured values at suitable measuring points in time in order to enable their applicability for the dentist. 

The effect of hypnosis on brain activity during the fMRI was shown by Halsband and Wolf [5]. Instead of a dental treatment, dental audio-visual stimuli as well as control stimuli were shown in a video presentation. This method has the advantage that it is more comparable than actual dental treatment. While individual factors and treatment options may differ in real dental treatment, each patient received the same stimulus. Based on a preliminary study, these stimuli were also tested for their effect and were therefore an adequate stimulus to simulate dental fear.

Although the fMRI examination method is difficult to quantify, the authors were able to present a before-and-after activity comparison of the brain regions, which led to adequate results [5]. The activities before and after hypnosis were compared. Since it was possible to obtain both test and control results from one patient, it can be assumed that the measurements were well comparable. Whether a video could be shown several times (before and after hypnosis), and thus, a certain anxiety-reducing adaptation effect could result, was not clear in the execution [5]. Possibly, a further comparison of the test group with a control group that also consists of anxiety patients and is not hypnotized could be another possibility to achieve good results.

The use of hypnosis in dentistry can be considered like the use of hypnosis medicine, such as in surgery. Invasive dental procedures are not uncommon, and surgical procedures such as extractions or implantations are daily routine procedures in the dental practice. Medical hypnosis, *i.e.*, the efficacy and safety of hypnosis techniques in somatic medicine, was investigated in a systematic review of meta-analyses of randomized clinical trials [34]. It could be determined that hypnosis is a safe and effective complementary technique for the use in medical procedures [34]. Suggestions in an awake state can also be part of an effective doctor–patient communication in daily clinical routine [34].

## 5. Conclusions

Our findings confirm that hypnosis shows positive effects for reducing dental anxiety and fear during dental treatment. It can be beneficial as an adjunct intervention that can be confirmed by patients. Many hypnosis strategies promise suitable approaches to counteract dental fear. However, despite positive effects of hypnotic interventions in the systematic review, the results of the meta-analysis are very heterogeneous. Unfortunately, there is no real measurable effect due to the heterogeneity of the only five studies included in the meta-analysis. There is a need to verify/determine the actual effects of hypnosis and a possible hypnosis-related intraoperative effect through further clinical studies. Within the limitations of the current study, a more consistent use of methods to examine anxiety for hypnosis research is recommended.

On the one hand, the results of the systematic review are promising and show that hypnosis can be considered an effective and successful method for anxiety reduction. However, on the other hand, the evaluation must also consider the results of the meta-analysis and the fact that there are study results showing no effect or even a slightly negative effect of hypnosis on reducing patients’ anxiety. These different and sometimes contradictory data should be further investigated.

## Figures and Tables

**Table 1:** General characteristics of the included studies. **AZI,** Aachen Dental Treatment Fear Inventory; **CCS**, case-control study; **Corah’s DAS/DAS-R,** Corah’s Dental Anxiety Scale/Revised DAS; **CT**, clinical trial; **DAS,** Dental Anxiety Scale; **DBS,** Dental Beliefs Survey; **DCQ,** Dental Cognitions Questionnaire; **DSR,** Dental Situation Reactions; **IDCI,** Revised Iowa Dental Control Index; **DFS,** Dental Fear Scale; **FLACC,** Face, Legs, Activity, Cry, Consolability; **FSS,** Fear Survey Schedule; **GFS^1^,** Geer Fear Scale; **GFS^2^,** Gatchel Fear Scale; **HAQ,** Hypnotic Attitudes Questionnaire; **HGSHS,** Harvard Group Scale of Hypnotic Susceptibility; **MACL,** Mood Adjective Check List; **PRDS,** Positive Reaction to Dentistry Scale; **RCT**, randomized clinical trial; **S-DAI,** Short Dental Anxiety Inventory; **SHCS,** Stanford Hypnotic Clinical Scale for adults; **STAI,** State-Trait Anxiety Inventory; **TAS,** Tellegen Absorption Scale; **VAS**, Visual Analogue Scale.

No.	Year	Author	Study Design	Scales	Quality Assessment
1	2017	Ramírez-Carrasco et al. [20]	RCT	FLACC	good
2	2015	Halsband and Wolf [5]	CT	DFS, HAQ, DAS-R, HGSH	good
3	2015	Glaesmer et al. [9]	RCT	VAS, DAS	good
4	2013	Abdeshahi et al. [7]	CCS	VAS, STAI	good
5	2011	Holdevici et al. [21]	CT	DFS	good
6	2011	Eitner et al. [22]	RCT	AZI	good
7	2011	Wannemueller et al. [17]	CT	DAS, DCQ, IDCI, STAI	good
8	2007	Di Clementi et al. [23]	CT	DAS, HGSHS, STAI, TAS	good
9	2006	Eitner et al. [24]	RCT	DAS, VAS, GFS^2^	good
11	2005	Hermes et al. [25]	CT	STAI	fair
10	2001	Willumsen et al. [26]	CT	DAS-R, DAS, DBS, DFS	good
12	2002	Moore et al. [15]	RCT	DAS, DFS, DBS, STAI, GFS^1^	good
13	2000	Ghoneim et al. [18]	RCT	STAI, VAS	good
14	1999	Aartman et al. [27]	CT	DAS, S-DAI	good
15	1997	Enqvist and Fischer [28]	RCT	VAS	good
16	1996	Moore et al. [16]	RCT	DAS, DBS, DFS, GFS^1^, VAS, STAI, SHCS	fair
17	1995	Lu et al. [29]	CT	DAS-R	fair
18	1995	Hammarstrand et al. [30]	RCT	DAS, DSR, GFS^1^, MACL P, MACL C	fair
19	1989	Gerschman et al. [31]	CT	DAS, DFS, FSS, PRDS, VAS	good

## Data Availability

All data related to the research are presented in the article.

## Supplementary Materials

The following supporting information can be downloaded at: https://www.mdpi.com/article/10.3390/brainsci12050521/s1, Supplementary Table S1: Details of included studies; Supplementary Table S2: List of excluded papers; Supplementary Table S3: PRISMA checklist.
