# Supplementary material for: Efficacy of Hypnosis on Dental Anxiety and Phobia: A Systematic Review and Meta-Analysis
Source: Brain Sci. 2022 Apr 20;12(5):521. doi: 10.3390/brainsci12050521 (PMC9138388; doi:10.3390/brainsci12050521)
Supplement: Supplementary file 1 [file brainsci-12-00521-s001.zip › Supplementary Table S1. Details of included studies.pdf]

Author	Type of study	Type of Study ocation	Number of subjects	M/F	Study length	Scores	Dental treatment /Study Design	hypnosis type/ Study design	outcomes
Ramírez-Carrasco et al.	RCT	Mexico	N=40<br>(5-9yy)	child ren	1 day	FLACC,<br>heart rate	dental care	2 groups<br>Hypnosis and control<br><br>-classic directive hypnosis intervention<br>3- minute progressive muscle relaxation induction followed by a 5-minute deepening procedure aimed at increasing the patients' focus, absorption, and concentration. (n=20)<br><br>-controlgroup without hypnosis (n= 20)	**FLACC :**<br>Hypnosis: 2.10<br>Control: 2.65<br>p-value = 0.5<br>The interrater reliability for the FLACC scale scoring in both observers yielded a Lin concordance correlation coefficient of 0.92 (CI 95%: 0.81–0.96)<br><br>**heart rate**<br>hypnosis at baseline: 94.16<br>control at baseline: 92.31<br>p-value:0 .7<br>hypnosis during LA: 99.3<br>control during LA93.57<br>p-value 0.2<br>A marginal statistical difference ($p = 0.05$) was found in the heart rate between<br>baseline and anesthetic moment,
Halsband & Wolf	CT	Germa ny	24<br>(19-57yy)	M/F	During the fMRI scan	DFS, HAQ,<br>DAS-R<br>HGSH	-	2 groups<br>-dental phobics (n=12)<br>Mean age 34.9<br>-healthy controls (n=12) mean age 33.15<br><br>Both groups<br>audio tape with hypnosis by Fiedler	Amygdala acitivity left before hypnosis<br>X= 18<br>Y=-10<br>Z=-12<br>t-value =5.23<br>p-value= <0.001<br>after hypnosis<br>x= -22<br>y=-7<br>z=-14<br>t-value= 4.13<br>p-value= <0.001<br>Phobics showed significant amygdala activity ($p < 0.001$)<br>during dental stimulation in the awake stage, after the hypnosis<br>no activity could be found there.

Glaesmer et al.	RCT	Germany	102<br>At the beginning 107 patients were included but 5 patients were excluded from the analyses because of the necessity of surgical access and osteotomy<br><br>Testgroup 51<br>Controlgroup 51	M/F	6 months	VAS, DAS	Tooth extraction	2 groups<br>-testgroup (TAU) standardized hypnosis CD before and during dental treatment into a trance state (n=51)<br><br>-controlgroup (n=51) (TAU +Hyp)<br>Without hypnosis	43,1% reported that hypnosis intensively reduced their anxiety during the treatment<br>VAS before treatment<br>TAU= 4.8 (SD2.6)<br>TAU+Hyp= 5.5 (SD 2.9)<br>p-value= .186<br><br>VAS during tooth extraction<br>TAU=3.6 (SD 2.6)<br>TAU+Hyp= 2.7 (SD 2.1)<br>p-value= 0.49<br>significant anxiety reduction during treatment<br><br>VAS after treatment<br>TAU= 2.0 (SD2.0)<br>TAU+Hyp= 1.7 (SD0.7)<br>P= .159
Abdeshahi et al.	CCS	Iran	N=24<br>(18-75yy)	M/F	Treatment duration	VAS for pain scoring STAI for anxiety scorin	Tooth extraction	2 groups (same patients)<br>-First treatment with standardized optical hypnosis method Chiassons tchnique *(N=24)*<br>-second treatment without hypnosis *(N=24)*	The results showed mean anxiety scores of<br><br>46.8 +- 3.8 and 47.4 +- 3.9 in the local anaesthesia<br><br>and hypnosis groups no statistically significant difference,<br><br>- Hypnosis: 2 (8.3%)<br>- LA: 8 (33.3%)<br>- P-value: 0.04

Eitner et al.	RCT	Germany	N=82 (19-80yy)	M/F	6 months	Bloodpressure, heartfrequency and Oxygen partial pressure before and during dental treatment AZI before treatment in testgroup 29.23 after treatment 15.29 in controlgroup 29.58 after treatment 30.15	Implant	2 groups<br>-Hypnotherapy per audio pillow *(N=44)*<br><br>-controlgroup (N=*38)*	In the hypnotherapy group (*n* = 44), the average score<br><br>before treatment was 29.23 ± 9.83 (range: 10–52);<br><br>after treatment, the average score was 15.29 ± 7.59 (range: 6–38)<br><br>In the control group, before treatment<br><br>was 29.58 ± 7.94 (range: 13–49); after treatment,<br><br>the average score was 30.18 ± 7.42 range (12–45)<br><br>The AZI scores for the hypnotherapy and control groups were compared using a parametric *t* test for average equality and the Levene test for equality of variance. Both methods showed a significant difference (*p* = .00014) between the groups.
Hermes et al.	CT	Germany	N=50 (hyp=25; control=25; f=24; m=26; <40 years=27; ≥ 40 years=23;: 10)<br>Age: total= 39,3±15,1;<br>hypnosis: 38,7±13,3;<br>control: 39,9±17,1	M/F	2 weeks for every proband	STAI Test one week before treatment, 15 minutes before treatment during treatment and 1 week after the treatment	surgical tooth extraction: 63; tooth extraction: 46; apicoectomy	2 groups<br>-testgroup standard hypnosis audiotape plus local anesthesia<br>*(N=25)*<br>-controlgroup *(N=25)*	-**Hypnosisgroup:**<br>Difference 11.28 (+-9.86)<br>Correlation 0.63 (p=0.001)<br>t-value 5.72 (p=0.000)<br>Female:<br>14.00 (±9.88) 0.55 (p=0.041) 5.30 (p=0.000)<br>male:<br>7.82 (±9.09) 0.75 (p=0.008) 2.85 (p=0.017)<br><40 Jahre:<br>8.08 (±9.54) 0.68 (p=0.011) 3.05 (p=0.010)<br>≥ 40 Jahre:<br>14.75 (±9.34) 0.65 (p=0.023) 5.47 (p=0.000)<br><br>**Controlgroup**<br>Difference 1.76 (+-8.51)<br>Correlation 0.82 (p=0.000)<br>t-value 1.03 (p=0.311)<br>Female:<br>5.10 (±7.13) 0.89 (p=0.001) 2.26 (p=0.050)<br>male:<br>-0.47 (±8.85) 0.79 (p=0.000) -0.2 (p=0.841)<br><40 Jahre:

									0.86 (±7.80) 0.86 (p=0.000) 0.41 (p=0.688)<br>≥ 40 Jahre:<br>2.91 (±9.60) 079 (p=0.004) 1.01 (p=0.338)
Ghonheim et al.	RCT	USA	N=60 patients for third molar tooth extraction (18-35y)	M/F	1 week before treatment until 3 days after the treatment	STAI, VAS, blood pressure, heart rate was measured<br><br>VAS experimental group: 15.8<br>VAS in controlgroup 16.3	Surgery, Extraction of third molars	2 groups<br>-experimental group<br>hypnosis tape and patients were instructed to listen to it every day for 1 week before surgery, including the morning of surgery (N=30)<br><br>-controlgroup without hypnosis N=30)	**STAI**<br><br>At baseline<br>-controlgroup (n=30)<br>30.8 (7.3)<br><br>-Experimentalgroup (n=30)<br>32.9 (8.7)<br><br>Immediate preoperative<br>-controlgroup (n=30)<br>Increase of STAI =11.7 (7.2)<br>➔ 42.5<br><br>-Experimentalgroup (n=30)<br>Increase STAI= 5.5 (13.9)<br>➔ 38.4<br>ased on a two-sided two-sample unequal variance t-test,<br>the means differed significantly (P = 0.03).

<table>
<tr><td>Enqvist & Fischer</td><td>RCT</td><td>Sweden</td><td>N=69</td><td>M/F</td><td>3 weeks before operation until operation</td><td>Visuelle Analogskala (VAS)</td><td>Extraction of third molars</td><td>2 groups
-Hypnosis with audiotape during 3 weeks before surgery for 20 min every day (N=33)
-control group (N=36)</td><td>VAS test
Anxiety at examination
-test group= 5
-control group= 2

Anxiety before surgery
-test group 4.4
-in control group 4.9

Wilcoxon signed rank test. p = .002 for control group; p = .05 for experimental group</td></tr>
<tr><td>Moore et al.</td><td>RCT</td><td>Denmark</td><td>N= 206
(19-65 yy)</td><td>M/F</td><td>3 years</td><td>DAS
Dental Fear Survey (DFS),
DentalBeliefsSurvey(DBS)
State–Trait AnxietyInventory(STAI)
A modified FSS-IIGeer FearScale(GFS)</td><td>Restaurative dentistry</td><td>2 groups
-hypnotherapy (HT) (N=22)
-group therapy (GT) (N=23)
-individual systematic desensitization(SD) (N=29)
-Jacobson's progressive muscle relaxation (N=26)

-controlgroup
(N=106)</td><td>Summary statistics for Dental Fear Survey (DFS) dental anxiety and Dental Beliefs Survey (DBS) dental beliefs/trust scale scores for intervention and reference control groups in chronological order (n = 171, See attrition flow diagram Fig. 1)
<table>
<tr><td></td><td></td><td colspan="12">Systematic desensitization (original n)</td></tr>
<tr><td></td><td></td><td colspan="2">Video (n = 27)</td><td colspan="2">Rehearsals (n = 33)</td><td colspan="2">Group Tx (n = 24)</td><td colspan="2">Hypnosis (n = 22)</td><td colspan="2">Control† (n = 65)</td><td colspan="2">Control 3 yr (n = 18)</td></tr>
<tr><td>Test</td><td>Time</td><td>Mean</td><td>SD</td><td>Mean</td><td>SD</td><td>Mean</td><td>SD</td><td>Mean</td><td>SD</td><td>Mean</td><td>SD</td><td>Mean</td><td>SD</td></tr>
<tr><td>DFS (20–100 points)</td><td></td><td></td><td></td><td></td><td></td><td></td><td></td><td></td><td></td><td></td><td></td><td></td><td></td></tr>
<tr><td>Pretest</td><td>T1</td><td>76.0</td><td>12.8</td><td>73.0</td><td>13.3</td><td>78.4</td><td>10.3</td><td>81.5</td><td>12.5</td><td>80.2</td><td>11.7</td><td>78.9</td><td>14.3</td></tr>
<tr><td>Post therapy</td><td>T2</td><td>39.5</td><td>12.8</td><td>37.8</td><td>10.5</td><td>36.5</td><td>10.3</td><td>38.1</td><td>14.9</td><td>*</td><td>*</td><td>*</td><td>*</td></tr>
<tr><td>Post dental Tx</td><td>T3</td><td>30.1</td><td>7.2</td><td>33.3</td><td>8.1</td><td>27.0</td><td>6.4</td><td>29.7</td><td>9.0</td><td>*</td><td>*</td><td>*</td><td>*</td></tr>
<tr><td>All at follow-up
(n at follow-up)</td><td>T4</td><td>41.1
(n = 24)</td><td>16.5</td><td>45.9
(n = 27)</td><td>16.0</td><td>36.0
(n = 19)</td><td>16.1</td><td>34.4
(n = 14)</td><td>12.6</td><td>66.7†
(n = 65)</td><td>21.0</td><td>74.2
(n = 18)</td><td>17.9</td></tr>
<tr><td>Regular attenders
(n at follow-up):</td><td>T4</td><td>38.3
(n = 19)</td><td>16.1</td><td>42.0
(n = 17)</td><td>13.6</td><td>31.6
(n = 16)</td><td>8.3</td><td>33.8
(n = 12)</td><td>19.6</td><td>52.5†
(n = 30)</td><td>19.6</td><td>66.3
(n = 7)</td><td>16.7</td></tr>
<tr><td>DBS (15–75 points)</td><td></td><td></td><td></td><td></td><td></td><td></td><td></td><td></td><td></td><td></td><td></td><td></td><td></td></tr>
<tr><td>Pretest</td><td>T1</td><td>46.6</td><td>13.6</td><td>49.6</td><td>9.9</td><td>46.6</td><td>11.5</td><td>48.3</td><td>13.5</td><td>47.3</td><td>14.7</td><td>43.9</td><td>15.2</td></tr>
<tr><td>Post therapy</td><td>T2</td><td>20.0</td><td>9.4</td><td>18.8</td><td>3.9</td><td>18.7</td><td>6.5</td><td>20.0</td><td>9.3</td><td>*</td><td>*</td><td>*</td><td>*</td></tr>
<tr><td>Post dental Tx</td><td>T3</td><td>16.8</td><td>3.8</td><td>18.0</td><td>3.9</td><td>16.2</td><td>2.2</td><td>16.3</td><td>2.8</td><td>*</td><td>*</td><td>*</td><td>*</td></tr>
<tr><td>All at follow-up
(n at follow-up)</td><td>T4</td><td>23.9
(n = 24)</td><td>9.9</td><td>24.4
(n = 27)</td><td>9.9</td><td>21.0
(n = 19)</td><td>9.4</td><td>21.2
(n = 14)</td><td>9.3</td><td>36.0†
(n = 65)</td><td>18.4</td><td>37.4
(n = 18)</td><td>18.1</td></tr>
<tr><td>Regular attenders
(n at follow-up)</td><td>T4</td><td>21.8
(n = 19)</td><td>9.7</td><td>19.5
(n = 17)</td><td>6.7</td><td>19.0
(n = 16)</td><td>5.3</td><td>21.3
(n = 12)</td><td>10.1</td><td>24.7†
(n = 30)</td><td>13.8</td><td>26.9
(n = 7)</td><td>16.7</td></tr>
</table>
*Not applicable.
†Observed mean = 5.9 yr.

DFS
there were significant reductions in dental anxiety in all groups
(P<0.001)
significant effect in DBS
(p<0.001)</td></tr>
<tr><td>Holdevici et al.</td><td>CT</td><td>Romania</td><td>N=44</td><td>M/F</td><td>2 years</td><td>DFS</td><td>Dental treatment</td><td>2 groups
-erickson's hypnotherapy (N=20)
-without hypnosis (N=24)</td><td>A significant difference was registered comparing post applying Ericksonians hypnosis techniques scores of the two groups, hence for the anxiety towards the dentist after the intervention, the score was (t (56) =-3,955, p>.001).

Also, as for the experimental group, a positive and significant correlation was found between the anxiety towards the dentist and the pain felt during the intervention: r(20)=0,382; p=0,037<0,05 bilateral</td></tr>
</table>

Wannemueller et al.	CT	Germany	N=137<br>77 completed the study<br>mean age = 38.5 years,	M/F	Begin of the study until one week after treatment	DAS, DCQ, IDCI, STAI	-	4 group design<br>-*(n=22)*Cognitive behavioral treatment<br>*Audiotape with relaxation instructions*<br><br>*(n=19)*Individualised Hypnosis<br><br>-*(n=22)*<br>Standard hypnosis CD with stan- dard hypnotic suggestions<br><br>-*(n=36)*General Anaesthesia with propofol	Separate group comparisons showed significantly greater improvement in the CBT compared to all other groups (CBT vs. IndHyp: $p<0.05$; CBT vs. StandHyp: $p<0.01$; CBT vs. GA: $p< 0.005$).<br>None of the other group comparisons showed significant differences

Moore et al.	RCT	Denmark	N=174<br>(18-85yy)	M/F	1 year	DAS,, DFS, Dental beliefs survey (DBS), STAI, modiefied Geer fear scale, VAS, Stanford hypnotic clinical scale (SHCS)	Restaurative dentistry	5 groups<br>-testgroup<br>SD= Jacobsons progressive muscle relaxation, -cassette tape *(N=68)*<br>-Grouptherapy<br>*(N=30)*<br>-Erickson hypnosis technique *(N=25)*,<br>-self hypnosis with audiotape and video desensitization during 1 year<br><br>-controlgroup *(N=51)*	Each experimental group showed a significant and meaningful reduction in dental anxiety (DFS) (P<0.001) and increased trust (DBS) (P<0.001) within groups as well as when compared with the waiting list group {P<0.001)<br>VAS scores decreased for all groups after test treatments (P<0.001)

Eitner et al.	RCT	Germany	45 highly anxiety and non anxiety patients<br>-20 for Implantsurgery 28 for control group	M/F	1,5 years	Parameters were EEG, ECG, heart rate, blood pressure, blood oxygen saturation, respiration rate, salivary cortisol concentration, and body temperature DAS, VAS, Gatchel fear scale	Implant-surgery	4 groups (A, B, C, D)<br>-Group A (n=13)<br>-Group B (n=7)<br>-Group C (n=8)<br>-Group D DAS >12 (n=9)<br>-Group D DAS< 12 (n=8)<br>-Group D all<br>monitoring hypnosis *(N=17)*<br>3 patients were excluded)<br><br>-3 (A, B, C) *(N=28)*	Group D was tested for DAS before and after treatment<br>DAS before hypnosis 4,6<br>VAS after Hypnosis 6,8 in testgroup<br>(0=feeling very bad – 10 feeling good)
Willumsen et al.	CT	Norway	N=65	M/F	10 treatments sessions during 10 weeks	CDAS, DFS, DBS<br>DAS	Restaurative dentistry	3 groups<br>CT = Cognitive Therapy Several cognitive and behavioral techni- ques *(N=21)*<br>-AR = Applied relaxation had two primary aims: to learn to recognize early signs of anxiety, and, second, to cope with this anxiety by initiating relaxation.<br>progressive relaxation by using a specially designed tape at home<br>During treatment they were instructed in progressive relaxation for 7min by therapist<br>*(N=21)*<br>-NO= Nitrous oxygen sedation<br>*(N=20)*	Scores on dental fear tests were significantly reduced compared with pretreatment level for all treatment groups.<br>There were no major differences between treatment methods in this short-term perspective<br>Multiple comparisons showed significant differences between CT and AR before treatment ($t = 2.26$, $P < 0.05$) and between NO and AR after treatment ($t = 2.36$, $P < 0.05$)

Hammarstrand et al.	RCT	Sweden	N=22<br>Mean age 31.8 yr,<br>n=8 were excluded	F	8 sessions	GFS, DAS, MACL P, MACL C, DSR(dental situations reaction) and GFS variables were measured pre- and posttreatment, respectively.	scaling, restoration of an upper jaw premolar or molar un- der local anesthesia, and restoration of an upper incisor under local anesthesia.	2 groups<br>-hypnotherapy (HT) (N=5) or a psychophysiologi- cal therapy(PP), progressive muscle relaxation (N=6)<br>-controlgroup without hypnosis (N=11)	The initial mean DAS score was 18.4 (SD 1.8), and 81.8% of the patients had a sum of scores between 18 and 20.<br>a significant reduction of fear as measured by the DAS, the DSR and a significant rise in the two mood dimensions.<br>Similar results were found for HT patients,
Lu et al.	CT	USA	N= 18<br>drug-dependet patients	M/F		Corah Dental Anxiety Scale all fit 13 or more only one had 12 before hypnosis or sedation, patient had to do eye roll test	restaurative dentistry, oral hygiene	1 group (N=18)<br>Hypnotic therapy<br>= patients were given repeated suggestions of relaxation<br>Or holding pencil and focus	With the combined hypnosedative approach, treatment outcomes were judged to be good or excellent in 11 of 18 patients.<br>They conclude that hypnosis can augment the effect so if sedation in this patient population
Gerschman et al.	CT	Australia	N=130 (5-60yy)<br>N=120<br>10 were excluded they were not hypnotizable	M/F	8 years	-VAS<br>-DAS<br>-DFS<br>the Fear Survey Schedule and a positive reaction to dentistry scale	Dental treatment	behavior therapy and cognitive therapy in association with hypnotherapy.	The most common fears were fear of not being able to express or cope with feelings of helplessness and defenselessness (87.7%),<br>fear of pain (86.2%),<br>fear of injections (84.6%),<br>generalized dental anxiety (78.5%),<br>fear of drilling (76.9%) and<br>fear of extractions (63.8%).<br>The majority of patients, 108 (83.1%),<br>had other phobic symptoms besides their dental phobia, i .e., multi- ple phobias.<br><br>The Chi-square analysis showed that the distribution of the scores of phobic dental patients were significantly different from that expected in the normal population ($X2 = 62.0$, $df = 2$ $p < 0.001$) and from the chronic pain patients, Table 2 ($X2 = 46$, $df = 2$ $p < 0.001$).

Di Clementi et al.	CT		N=291	M/F	-	DAS , STAI, HGSHS, Tellegen Absorption scale	a video of a dental procedure	2 studies were compared with 2 groups<br>-one group hypnosis with tape recording<br>-controlgroup without hypnosis	A two-way analysis of variance showed a marginal effect for hypnosis ($F_{1,290}$ = 3.20, P = .07),<br>The authors found an effect for group on the DAS (F1,228 = 3.67, P = .057),

Aartman et al.	CT	Netherland	N=211<br>(67 did not receive restorative dental treat- ment in the clinic for several reasons)<br><br>N=144<br><br>(17-69yy)	M/F	1 year	DAS, S-DAI (short dental anxiety inventory)	Restaurative dentistry	3 groups:<br>behavioural management approach(tell show do technique) (BM) (N=67),<br>nitrous oxide sedation (NOS)(N=40),<br>intravenous sedation IVS) (N=33),<br>Gerneral anasthesia (N=4).	With regard to DAS and S-DAI scores, there were<br>no statistically significant differences between the BM, NOS, and IVS groups found ($P > 0.05$)<br>no statistically significant with regard to SCL-90 total score and its subscales ($P > 0.05$)
