# Supplementary material for: Efficacy of Hypnosis on Dental Anxiety and Phobia: A Systematic Review and Meta-Analysis
Source: Brain Sci. 2022 Apr 20;12(5):521. doi: 10.3390/brainsci12050521 (PMC9138388; doi:10.3390/brainsci12050521)
Supplement: Supplementary file 1 [file brainsci-12-00521-s001.zip › Supplementary Table S2. List of excluded papers.pdf]

ID	*Author*	*Year*	*Sources*	*reason for exclusion*
1	Park et al,.	2019	Eur J Oral Sci. 2019 Feb;127(1):45-51	non pertinent
2	Torun et al,.	2017	Int J Oral Maxillofac Surg. 2017 Mar;46(3):379-384	non pertinent
3	Dantas et al,.	2017	Med Oral Patol Oral Cir Bucal. 2017 Jan 1;22(1):	non pertinent
4	Ohkushi et al,.	2016	Anesth Prog. 2016 Winter;63(4):175-180	non pertinent
5	Di Nasso et al,.	2016	J Endod. 2016 Sep;42(9):1338-43	non pertinent
6	Conway et al,.	2016	Cochrane Database Syst Rev	non pertinent
7	Kim et al,.	2015	Anesth Prog	non pertinent
8	Dunlop, D.	2015	J. Psychotherapy and Psychosomatics	non pertinent
9	De Morais et al,.	2015	J. of Oral Maxillofac Surg	non pertinent
10	Yu et al,.	2014	J Oral Maxillofac Surg. 2014 Sep;43(9):1148-53	non pertinent
11	Smiley et al,.	2014	Anesth Prog. 2014 Spring;61(1):3-10	non pertinent
12	Setty et al,.	2014	J Endod **40**(9): 1327-1331.	non pertinent
13	Robb, N.	2014	Br Dent J. 2014 Mar;216(5):223-7	non pertinent
14	Morino et al,.	2014	J Prosthodont Res. 2014 Jul;58(3):177-83	non pertinent
15	Pereira-Santos et al,.	2013	J Craniofac Surg	non pertinent
16	Montagnese et Setty.	2013	J Endod **39**(3): 415-416.	non pertinent
17	Lu et Lu,.	2013	Int J Clin Exp Hypn **61**(3): 271-281.	non pertinent
18	Jaggi et al,.	2013	J Contemp Dent Pract **14**(4): 605-609.	non pertinent
19	Fan et al,.	2013	Br J Oral Maxillofac Surg **51**(5): 428-433.	non pertinent
20	Collade et al,.	2013	PLoS One **8**(8): e71240.	non pertinent
21	Armfield et al,.	2013	Aust Dent J **58**(4): 390-407; quiz 531.	non pertinent
22	Studer et al,.	2012	Oral Maxillofac Surg **16**(4): 341-347	non pertinent
23	Romano et al,.	2012	Clin Oral Implants Res **23**(4): 489-495.	non pertinent
24	Munoz et al,.	2012	Int J Oral Maxillofac Implants **27**(2): 375-382.	non pertinent
25	Miyawaki et al,.	2012	Immunopharmacol Immunotoxicol **34**(1): 79-83.	non pertinent
26	Hennequin et al,.	2012	Clin Oral Investig **16**(2): 633-642.	non pertinent
27	Huet et al,.	2011	International journal of clinical and experimental hypnosis **59**(4): 424-440.	non pertinent
28	Boyd et al,.	2011	J Oral Maxillofac Surg **69**(6): 1608-1612.	non pertinent
29	Abdullah et al,.	2010	Aust Dent J **56**(3): 296-301.	non pertinent
30	Tsugayasu et al	2010	J Oral Maxillofac Surg **68**(3): 590-596.	non pertinent
31	Okawa et al,.	2010	Anesth Analg **110**(2): 415-418.	non pertinent
32	Manani et al,.	2010	Minerva Stomatol **59**(9): 489-506.	non pertinent
33	Lu, D.P	2010	General dentistry **58**(3): e140-e147	non pertinent
34	Peltier, B.	2009	Special Care in Dentistry **29**(1): 51-57.	non pertinent
35	Nutt et al,.	2009	J Psychopharmacol **23**(8): 867-873.	non pertinent
36	Larentsova et al,.	2009	Bull Exp Biol Med **148**(2): 340-342.	non pertinent
37	Maeda et al,.	2008	Anesth Prog **55**(3): 73-77	non pertinent
38	Lindenmann et al,.	2008	J Endod **34**(10): 1167-1170.	non pertinent
39	Lee et al,.	2008	J Oral Maxillofac Surg **66**(10): 1996-2003.	non pertinent
40	Manani et al,.	2007	Minerva Stomatol **56**(3): 85-104.	non pertinent
41	Karst et al,.	2007	Anesth Analg **104**(2): 295-300.	non pertinent
42	Faulks et al,.	2007	Dev Med Child Neurol **49**(8): 621-625.	non pertinent
43	Coldwell et al,.	2007	J Anxiety Disord **21**(7): 871-887.	non pertinent
44	Jackson et al,.	2006	J Clin Psychopharmacol **26**(1): 4-8.	non pertinent
45	Collini et al,.	2006	Minerva Stomatol **55**(3): 99-113.	non pertinent

46	Vallejo et al,.	2005	J Clin Anesth **17**(7): 543-548.	non pertinent
47	Manani et al,.	2005	Minerva Stomatol **54**(10): 551-568.	non pertinent
48	Jerjes et al,.	2005	Oral Surg Oral Med Oral Pathol Oral Radiol Endod **100**(5): 564-570.	non pertinent
49	Ganzberg et al,.	2005	Anesth Prog **52**(4): 128-131.	non pertinent
50	Fong et al,.	2005	Anaesth Intensive Care **33**(1): 73-77.	non pertinent
51	De Jongh et al,.	2005	Int Dent J **55**(2): 73-80.	non pertinent
52	Schaira et al,.	2004	Anesth Prog **51**(1): 14-18.	non pertinent
53	Quernstorm et al,.	2004	Gen Dent **52**(6): 496-501.	non pertinent
54	Manani et al,.	2004	Minerva Stomatol **53**(5): 241-250.	non pertinent
55	Leitch et al,.	2004	Anaesthesia **59**(9): 853-860.	non pertinent
56	Goodchild et al,.	2004	Gen Dent **52**(3): 264-268;	non pertinent
57	Bavisha et al,.	2004	Eur J Anaesthesiol **21**(4): 284-288.	non pertinent
58	Wolf et al,.	2003	J Clin Psychopharmacol **23**(1): 51-57.	non pertinent
59	Willumsen et al,.	2003	Acta Odontol Scand **61**(2): 93-99.	non pertinent
60	Willemsen, R.	2003	Revue belge de médecine dentaire. Belgisch tijdschrift voor tandheelkunde **58**(2): 99-104.	non pertinent
61	Suzuki et al,.	2003	Eur J Clin Pharmacol **58**(12): 829-833.	non pertinent
62	Finkelstein et al,.	2003	Int J Clin Exp Hypn **51**(1): 77-85.	non pertinent
63	Faraco et al,.	2003	Braz Dent J **14**(3): 215-219.	non pertinent
64	Aeschliman et al,.	2003	J Periodontol **74**(7): 1056-1059.	non pertinent
65	Ackley, D. C.	2003	Dentistry Today **22**(1): 96-102.	non pertinent
66	Wilner et al,.	2002	J Clin Psychopharmacol **22**(2): 206-210.	non pertinent
67	Robin, C. et Trieger N.	2002	Anesth Prog **49**(4): 128-132.	non pertinent
68	Noble, S.	2002	Dental update **29**(2): 70-74.	non pertinent
70	Jackson et al,.	2002	Dent Clin North Am **46**(4): 767-780.	non pertinent
71	Jackson et al,.	2002	Dent Clin North Am **46**(4): 781-802.	non pertinent
72	Girdler et al,.	2002	Anaesthesia	non pertinent
73	Willumsen et al,.	2001	Acta Odontol Scand	non pertinent
74	Speirs et al,.	2001	Br Dent J **190**(8): 444-449.	non pertinent
75	Thom et al,.	2000	J Consult Clin Psychol **68**(3): 378-387.	non pertinent
76	Tan et al,.	2000	Singapore Dent J **23**(1 Suppl): 18-22.	non pertinent
77	Lu et al,.	2000	Gen Dent **48**(4): 446-452.	non pertinent
78	Koerner et al	2000	for the apprehensive dental patient." Dent Today **19**(4): 96, 98-101	non pertinent
79	Johren et al,.	2000	Br J Oral Maxillofac Surg **38**(6): 612-616.	non pertinent
80	Girdler et al,.	2000	Anaesthesia **55**(4): 327-333	non pertinent
81	Craig et al,.	2000	J Clin Periodontol **27**(12): 955-959.	non pertinent
82	Benson et al,.	2000	Ann R Australas Coll Dent Surg **15**: 284-285.	non pertinent
83	Bell et al,.	2000	Br J Oral Maxillofac Surg **38**(6): 596-602.	non pertinent
84	Thompson et al,.	1999	Br Dent J **187**(10): 557-562	non pertinent
85	Smyth, J. S	1999	Aust Dent J **44**(4): 275-278.	non pertinent
86	Oshima et al	1999	Br J Anaesth **82**(5): 698-702.	non pertinent
87	Moore et al,.	1999	J Am Dent Assoc **130**(4): 541-554.	non pertinent
88	Lyons, H.	1999	SAAD Dig **16**(4): 3-7	non pertinent
89	Homma et al,.	1999	Br J Anaesth **82**(4): 570-574.	non pertinent
90	Crecelus	1999	Anesth Prog **46**(3): 100-103.	non pertinent
91	Parwoth et al,.	1998	J Oral Maxillofac Surg **56**(4): 447-453	non pertinent
92	Oei-Lim et al,.	1998	Anesth Analg **86**(5): 967-972	non pertinent

93	Miller et al,.	1998	Anesth Prog **45**(2): 68-73.	non pertinent
94	Mamiya et al,.	1998	Anesth Prog **45**(1): 18-21.	non pertinent
95	Coldwell et al,.	1998	Pharmacol Biochem Behav **59**(2): 537-545	non pertinent
96	Coldwell et al,.	1998	Behav Res Ther **36**(4): 429-441	non pertinent
97	Bennett et al,.	1998	Oral Maxillofac Surg **56**(11): 1249-1254.	non pertinent
98	Singer et Dionne.	1997	J Orofac Pain **11**(2): 139-146.	non pertinent
99	Primosch et al,.	1997	Pediatr Dent **19**(8): 480-483	non pertinent
100	Nakanishi et al,.	1997	Oral Surg Oral Med Oral Pathol Oral Radiol Endod **84**(1): 11-15.	non pertinent
101	Moore et al,.	1997	Oral Surg Oral Med Oral Pathol Oral Radiol Endod **84**(1): 5-10.	non pertinent
102	Matsushima et al,.	1997	Biol Psychiatry **41**(12): 1211-1217.	non pertinent
103	Gear et al,.	1997	Pain **71**(1): 25-29	non pertinent
104	Coldwell et al,.	1997	J Oral Maxillofac Surg **55**(10): 1061-107.	non pertinent
105	Berthold et al,.	1997	Oral Surg Oral Med Oral Pathol Oral Radiol Endod **84**(2): 119-124.	non pertinent
106	Tobey, H. S	1996	J N J Dent Assoc **67**(4): 21-24.	non pertinent
107	Peretz et al,.	1996	International Dental Journal **46**(2): 108-112.	non pertinent
108	Luotio et al,.	1996	Oral Surg Oral Diagn **7**: 15-19	non pertinent
109	Fabian, T.; et Zelles, T.	1996	Journal of dental research **75**	non pertinent
110	Burstein et al,.	1996	Anesth Prog **43**(2): 52-57.	non pertinent
111	Biron, C.R	1995	Rdh **16**(11): 42-44, 46	non pertinent
112	Manani et al,.	1995	Anesth Prog **42**(3-4): 107-112.	non pertinent
113	Herod et al,.	1995	Gen Dent **43**(3): 267-269	non pertinent
114	Brown et al,.	1995	J Nerv Ment Dis **183**(9): 603-604.	non pertinent
115	Van der Bijl et al,.	1994	Ann Dent **53**(1): 37-38.	non pertinent
116	Rustvold, S.R.	1994	General dentistry **42**(4): 346-348	non pertinent
117	Foelofse et al,.	1994	J Oral Maxillofac Surg **52**(3): 247-250.	non pertinent
118	Oster, M.I.	1994	Am J Clin Hypn **37**(1): 12-21.	non pertinent
119	Oakley et al,.	1994	Journal of orofacial pain **8**(4): 397-401.	non pertinent
120	Milgrom et al,.	1994	J Oral Maxillofac Surg **52**(3): 219-224;	non pertinent
121	Milgrom et al,.	1994	Anesth Prog **41**(3): 70-	non pertinent
122	Kaufman et al,.	1994	J Oral Maxillofac Surg **52**(8): 840-843	non pertinent
123	Gokli et al,.	1994	ASDC journal of dentistry for children **61**(4): 272-275.	non pertinent
124	Fukuta et al,.	1994	J Clin Pediatr Dent **18**(4): 259-265.	non pertinent
125	DAvila et al,.	1994	ASDC J Dent Child **61**(4): 276-281.	non pertinent
126	Barsby, M. J.	1994	Br Dent J **176**(3): 97-102	non pertinent
127	Stopperich et al,.	1993	Anesth Prog **40**(4): 117-121	non pertinent
128	Richards et al,.	1993	Oral Surg Oral Med Oral Pathol **76**(4): 408-411.	non pertinent
129	Migrom et al,.	1993	Anesth Prog **40**(3): 57-62.	non pertinent
130	Mazey et al,.	1993	J Calif Dent Assoc **21**(3): 17-25.	non pertinent
131	Litt et al	1993	J Dent Res **72**(8): 1237-1243	non pertinent
132	Brody, H. A.	1993	J Calif Dent Assoc **21**(3): 31-32, 34.	non pertinent
133	Skelly et al,.	1992	Br Dent J **172**(4): 153-157.	non pertinent
134	Van der Bijl et al,.	1991	Oral Maxillofac Surg **49**(7): 672-678;	non pertinent
135	Finkelstein, S.	1991	Am J Clin Hypn **33**(3): 187-191.	non pertinent
136	Rodolfa et al,.	1990	American Journal of Clinical Hypnosis **33**(1): 22-28.	non pertinent
137	Moore et al,.	1990	Anesth Prog **37**(6): 308-311.	non pertinent
138	Kryshtalskyj et al,.	1990	Oral Surg Oral Med Oral Pathol **69**(4): 413-419.	non pertinent
139	Kallio et al,.	1990	Acta Anaesthesiol Scand **34**(3): 171-175.	non pertinent

140	Richmond et al,.	1988	Anaesthesia **43**(8): 694-696.	non pertinent
141	O`Boyle et al,.	1988	Br J Anaesth **60**(4): 419-425.	non pertinent
142	Luyk et al,.	1988	Int J Oral Maxillofac Surg **17**(6): 347-3	non pertinent
143	Hosie et al,.	1988	Br J Anaesth **60**(1): 18-23.	non pertinent
144	Hallonsten, A. L.	1988	Acta Anaesthesiol Scand Suppl **88**: 27-30.	non pertinent
145	Forgione et al,.	1988	Dental Clinics of North America **32**(4): 745-761.	Abstract only
146	Dolan et al,.	1988	Oral Surg Oral Med Oral Pathol **66**(5): 536-539.	non pertinent
147	Dolan et al,.	1988	J Oral Maxillofac Surg **46**(6): 471-473.	non pertinent
148	Van der Bijl et al,.	1987	Int J Oral Maxillofac Surg **16**(3): 325-332.	non pertinent
149	O' Boyle et al,.	1987	Br J Anaesth **59**(6): 746-754.	non pertinent
150	O`Boyle et al,.	1987	Psychopharmacology (Berl) **91**(2): 244-247.	non pertinent
151	Harris et al,.	1987	Br Dent J **162**(8): 297-301.	non pertinent
152	Schutt et al,.	1986	Dent Clin North Am **30**(4 Suppl): S93-105	non pertinent
153	Lundgren et al,.	1986	Int J Oral Maxillofac Surg **15**(5): 541-548	non pertinent
156	Barker et al,.	1986	Br Dent J **157**(2): 68.	non pertinent
157	Renouf, D.	1985	General dentistry **33**(1): 62-6	Abstract only
158	Morcas, B. A.	1984	Br Dent J **157**(2): 68.	Abstract only
159	Baker, S. R. et Boaz, D.	1983	Int J Clin Exp Hypn **31**(1): 14-18.	non pertinent
160	Yamauchi, K.T.	1981	Am J Clin Hypn **24**(2): 128-131.	non pertinent
161	Biron, G.E.	1981	J Am Soc Psychosom Dent Med **28**(4): 118-121.	non pertinent
162	Rubin et al,.	1980	S Afr Med J **58**(3): 124-126.	non pertinent
163	Lambert, C.	1980	Aust Dent J **25**(2): 81-83.	non pertinent
164	Kelly, S. F.	1980	Int J Clin Exp Hypn **28**(3): 189-191.	non pertinent
165	Hovi-Viander et al,.	1980	J. Oral Surg 38; 3	non pertinent
166	Donaldson et al,.	1980	Anesth Prog **27**(1): 18-20.	non pertinent
167	Dixon et al,.	1980	Br J Anaesth **52**(5): 517-526.	non pertinent
168	Barclay et al,.	1980	Br J Oral Surg **18**(2): 141-149.	non pertinent
169	Litchfield, N. B.	1979	Dent Anaesth Sedat **8**(2): 87-90.	non pertinent
170	Halsband & Wolf	2019	Int. J. of Clinical and Experimental Hypnosis 67, 449-474	non pertinent
171	De Stefano et al,.	2019	Minerva Stomatol ; 68(6): 317-331, 2019 Dec.	non pertinent
172	Arabzade Moghadam S et al,.	2021	Clinical and experimental dental research, June 2021	Non pertinent
173	Diercke et al,.	2013	*J Health Psychol 2013 18: 1519*	non pertinent
174	Hill et al,.	2008	*British Dental Journal,* **2008**, E13 1-5	non pertinent
175	Dailey et al,.	2001	*British dental journal* 190 April **2001,** 450-453	non pertinent
176	Enkling et al,.	2006	*Clin Oral Invest* **2006** 10: 84–91	non pertinent
177	Facco et al,.	2014	*Minerva Anestesiologica Vol. 79, Nr 12* **2014** 1389-1395	non pertinent
178	Fábián & Fàbiàn	*1998*	*ANNALS NEW YORK ACADEMY OF SCIENCES 495-500*	non pertinent
179	Hakeberg et. Berggren	1997	*Acta Odontol Scand,* **1997**, 55, 314-318	non pertinent
180	Hermes et al,.	2005	*Journal of Cranio-Maxillofacial* Surgery, **2005,** 33, 123–129	non pertinent
181	Eitner S et al.	2006	*Intl. Journal of Clinical and Experimental Hypnosis*, 54(4): 457–479, **2006**	non pertinent
182	Katcher et al,.	*1984*	*American Journal of clinical hypnosis, VOLUME 27. NUMBER I. JUL Y* ***1984***	non pertinent
183	Montenegro et al,.	2017	*American Journal of Clinical Hypnosis*, 59, **2017**, 414-421	non pertinent
184	Gottlieb M.	*2011*	*Today's FDA, January/February* ***2011***	non pertinent
185	Bar-Gil B. et al.,	*1983*	*J of the American Society of Psychosomatic Dentistry and Medicine* February, **1983**	non pertinent

186	Morse et al,.	*1983*	*Journal of Human Stress, 7:3,* ***1983*** *19-26*	non pertinent
187	Ferdeghini et al,.	*2018*	*Journal of biological regulators and homeostatic agents,* ***2018,***	Non pertinent
188	Meyerson et Uziel	*2014*	*Intl. Journal of Clinical and Experimental Hypnosis*, 62(2): 179–187, 2014	Non pertinent
